# H-intensity scale score to estimate CSF GluN1 antibody titers with one-time immunostaining using a commercial assay

**DOI:** 10.3389/fimmu.2024.1350837

**Published:** 2024-04-30

**Authors:** Masaki Iizuka, Naomi Nagata, Naomi Kanazawa, Tomomi Iwami, Makoto Nagashima, Masaaki Nakamura, Juntaro Kaneko, Eiji Kitamura, Kazutoshi Nishiyama, Noritaka Mamorita, Takahiro Iizuka

**Affiliations:** ^1^ Department of Neurology, Kitasato University School of Medicine, Sagamihara, Japan; ^2^ Department of Medical Informatics, Kitasato University School of Allied Health Sciences, Sagamihara, Japan

**Keywords:** NMDA receptor encephalitis, immunohistochemistry, autoantibodies, cell-based assay, tissue-based assay

## Abstract

**Introduction:**

Anti-NMDA receptor encephalitis is an autoimmune disorder caused by autoantibodies (abs) against the conformational epitope on GluN1 subunits. GluN1-abs have been determined with cell-based assay (CBA) co-expressing GluN1/GluN2 subunits. However, commercial fixed CBA expressing only GluN1 subunit has increasingly been used in clinical practice. The ab titers can be determined with serial dilutions, but its clinical significance remains unclear. We aimed to develop an H-intensity scale (HIS) score to estimate GluN1-ab titers in cerebrospinal fluid (CSF) with one-time immunostaining using both commercial CBA and immunohistochemistry and report its usefulness. “H” is the initial of a patient with high CSF GluN1-ab titers (1:2,048).

**Methods:**

We first determined the reliability of CBA in 370 patients with suspected autoimmune encephalitis by comparing the results between commercial CBA and established assay in Dalmau’s Lab. Then, we made positive control panels using the patient H’s CSF diluted in a fourfold serial dilution method (1:2, 1:8, 1:32, 1:128, 1:512, and 1:2,048). Based on the panels, we scored the intensity of ab reactivity of 79 GluN1-ab-positive patients’ CSF (diluted at 1:2) on a scale from 0 to 6 (with ≥1 considered positive). To assess inter-assay reliability, we performed immunostaining twice in 21 patients’ CSF. We investigated an association between the score of CSF obtained at diagnosis and the clinical/paraclinical features.

**Results:**

The sensitivity and specificity of CBA were 93.7% (95% CI: 86.0–97.3) and 98.6% (95% CI: 96.5–99.5), respectively. Linear regression analysis showed a good agreement between the scores of the first and second assays. Patients with a typical spectrum, need for mechanical ventilation support, autonomic symptoms/central hypoventilation, dyskinesias, speech dysfunction, decreased level of consciousness, preceding headache, ovarian teratoma, and CSF leukocyte count >20 cells/µL had a higher median HIS score than those without, but HIS score was not associated with sex, age at onset, or seizure. HIS score at diagnosis had a significant effect on 1-year functional status.

**Discussion:**

The severity of disease and four of the six core symptoms were associated with higher GluN1-ab titers in CSF at diagnosis, which may play a role in poor 1-year functional status. An incomplete phenotype can be attributed to low CSF GluN1-ab titers.

## 1 Introduction

Autoimmune encephalitis (AE) is defined as a form of encephalitis that occurs as a result of a brain-specific immune response, and it usually associates with autoantibodies (abs) against a neuronal or glial cell surface antigen (1). Anti-NMDA receptor (NMDAR) encephalitis is one of the most common AE characterized by viral prodrome followed by memory or psychobehavioral alterations, seizures, decreased level of consciousness, dyskinesias, speech dysfunction, autonomic symptoms, and central hypoventilation or a combination of these symptoms (2–4). Anti-NMDAR encephalitis is caused by abs against the conformational epitope on the extracellular amino terminal domain of the GluN1 subunit (GluN1-abs) (5). A definite diagnosis requires confirmation of the presence of GluN1-abs in cerebrospinal fluid (CSF) with appropriate assay (6).

The GluN1-abs have been determined with live or fixed cell-based assay (CBA) co-expressing GluN1/GluN2 subunits of the NMDAR in the research laboratory (1–4). However, in clinical practice, commercial fixed CBA expressing only GluN1 subunit has increasingly been used. Ab titers can be determined with ELISA (7) or serial dilutions in the commercial laboratory for a fee, but serial dilutions result in an increase in the cost, making it difficult to determine ab titers in many patients in clinical practice. Previous studies (7–10) reported an association between CSF GluN1-ab titers at diagnosis and some of the clinical features, but its clinical significance remains unclear.

To resolve these issues, we aimed to develop an intensity-based scale score to estimate CSF ab titers without serial dilutions and assessed whether the score of CSF obtained at diagnosis is associated with certain clinical and paraclinical features of this disorder. We named the score as “H-intensity scale (HIS) score”, in which the “H” is the initial of a patient whose CSF containing high ab titers (1:2,048) was used to make positive control panels to score the intensity of ab reactivity. We used the name “HIS score” after consent from the patient H who had achieved full recovery.

## 2 Materials and methods

### 2.1 Patient selection and antibody measurement

First, we retrospectively reviewed the clinical information of 710 patients with suspected AE or related disorder who underwent testing for neuronal surface (NS)-abs between January 1, 2007 and September 30, 2023. Among those, 470 (66.2%) patients’ CSF/sera were referred from other 163 hospitals to Kitasato University to examine NS-abs. The detailed clinical information was provided from each physician to TI (a principal investigator). The inclusion criteria of this cohort are as follows: AE or related neurological disorder is highly suspected based on clinical assessment; detailed clinical information is available for review by TI, including the mode of onset of symptoms, past history, family history, regular medications, neurologic examination, neuropsychological assessment, laboratory test results (blood, CSF, electro-encephalography, brain or spinal MRIs, and body CT), and subsequent clinical course and outcome when available; and written informed consent is obtained from the patients or their proxies.

In all patients, NS-abs were measured at the laboratory of Josep Dalmau (Dalmau’s Lab at University of Pennsylvania, Philadelphia, or IDIBAPS Hospital Clinic, Barcelona) because of it being one of the most advanced research laboratories, where many of the novel NS-abs have been identified since the first discovery of NMDAR-abs in 2007 (2). NS-abs were determined with previously established assays, such as rat brain immunohistochemistry (IHC) adapted to NS antigens and CBA (2, 11–18). Live neurons were also added when needed to determine the presence of NS-abs. The NS antigens examined included the NMDAR, α-amino-3-hydroxy-5-methyl-4-isoxazolepropionic acid receptor (AMPAR), γ-aminobutyric acid A receptor (GABAaR), γ-aminobutyric acid B receptor (GABAbR), glutamate kainate receptor subunit 2 (GluK2), metabotropic glutamate receptor 5 (mGluR5), metabotropic glutamate receptor 1 (mGluR1), dipeptidyl peptidase-like protein 6 (DPPX), contactin-associated protein-like 2 (Caspr2), leucine-rich glioma-inactivated 1 (LGI1), neurexin 3, or glycine receptor (GlyR). These antigens were examined based on the clinical phenotypes and/or immunoreactivity pattern on in-house IHC. Abs against myelin oligodendrocyte glycoprotein (MOG), aquaporin-4 (AQP4), or glial fibrillary acidic proteins (GFAP) were also examined in CSF and/or serum with established CBA (19), when being clinically or radiologically suspected, or based on immunoreactivity pattern on in-house or commercial IHC.

We also retrospectively examined NS-abs in the archived CSF at Kitasato University in appropriately half of the patients with commercial fixed CBA for NMDAR or others, commercial rat brain IHC, and/or in-house IHC adapted to NS antigens, as previously reported elsewhere (20).

### 2.2 Part I (reliability of commercial fixed CBA expressing only GluN1 subunit)

**Figure 1:**
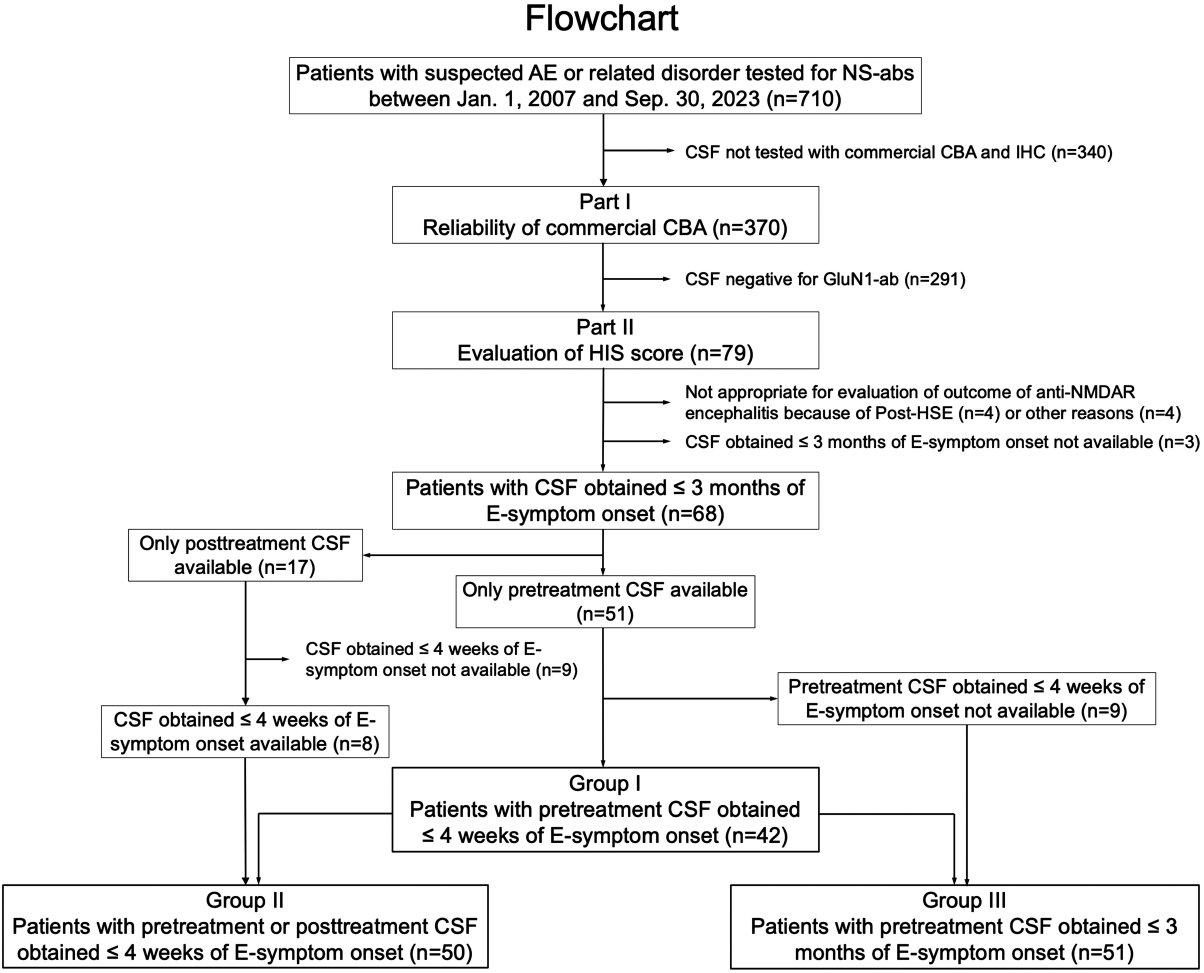
Flowchart. This figure shows a diagram of the study conducted. In part I, the reliability of a commercial fixed cell-based assay (CBA) expressing only GluN1 subunits was investigated, while in part II, the clinical significance of an H-intensity scale (HIS) score was evaluated in three overlapping groups. The number of patients is shown in parentheses. AE, autoimmune encephalitis; ab, antibodies; E-symptom, encephalitis symptom (see Text); NS, neuronal surface; post-HSE, autoimmune post-herpes simplex encephalitis.

In part I, we included 370 patients whose residual archived samples of the CSF identical to one examined at Dalmau’s Lab were available for this study (**Figure 1**). GluN1-abs were examined with a commercial kit (EUROIMMUN AG, product no.: FA 111m-3, Lübeck, Germany) at Kitasato University to evaluate the reliability of the commercial CBA. The kit consists of four biochips per field, containing the NMDAR (only GluN1 subunits)-transfected cells, control-transfected cells, hippocampus, and cerebellum, as previously reported (20).

In this study, we evaluated the intensity of ab reactivity of the CSF diluted at 1:2 instead of non-diluted CSF because we usually use CSF diluted at 1:2 for screening of NS-abs (20) on in-house IHC or to determine estimated CSF ab titers; otherwise, we followed the instruction of the company with indirect immunofluorescence assay (IIFA). The ab reactivity on CBA and IHC was evaluated with an Olympus BX53 fluorescence microscope equipped with a DP80 digital camera, CellSence Dimension1.8 imaging Software (Olympus, Japan). Green fluorescence was detected using a fluorescein-isothiocyanate filter (excitation: 470–495 nm, emission: 510–505 nm, and dichroic: 505 nm; U-FBNA, Olympus, Japan).

Ab reactivity to commercial fixed CBA was initially evaluated by three authors (NN, NK, and TI) independently and was finally determined by agreement of the authors. GluN1-ab-positivity or negativity was determined based on the results performed at Dalmau’s Lab, and the sensitivity and the specificity of the commercial CBA were calculated.

### 2.3 Part II (evaluation of HIS score)

#### 2.3.1 Development of positive control panels

To make positive control panels, we first selected patient H who presented with a typical spectrum of anti-NMDAR encephalitis, whose CSF ab titers were thought to be highest among patients who had been examined with the commercial CBA.

**Figure 2:**
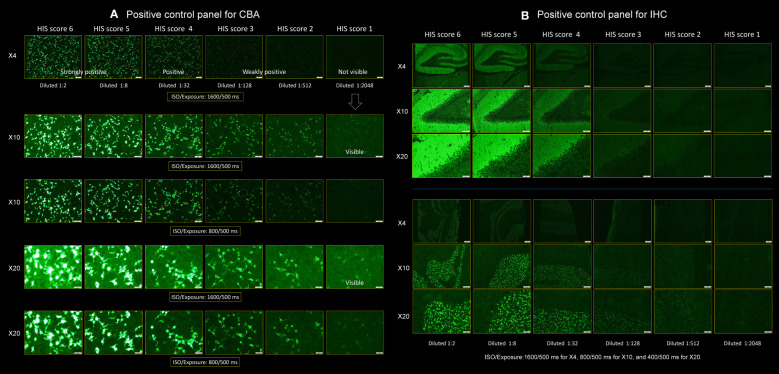
Positive control panels for CBA and IHC. This figure shows a pair of positive control panels. Each panel was made using the cerebrospinal fluid (CSF) of a “patient H” having ab titers of 1:2,048, which was diluted in a fourfold serial dilution method, ranging from 1:2, 1:8, 1:32, 1:128, and 1:512 to 1: 2,048 to score the individual patient’s CSF. **(A)** Positive control panel for CBA and **(B)** positive control panel for IHC (upper: hippocampus, lower: cerebellum). A series of photographs was taken in several settings by adjusting the ISO/Exposure. In **(A)**, the photographs were taken under the two settings of ISO/Exposure (1,600/500 and 800/500 ms) when viewing the slide under ×4, ×10, and ×20 objective lens. In **(B)**, the photographs were taken under the three settings of ISO/Exposure adjusted for each magnification of the objective lens as follows: 1,600/500 ms for ×4, 800/500 ms for ×10, and 400/500 ms for ×20, respectively (see text). CBA, cell-based assay; HIS, H-intensity scale; IHC, immunohistochemistry.

Patient H’s CSF was diluted in a fourfold serial dilution method (1:2, 1:8, 1:32, 1:128, 1:512, and 1:2,048) to make the positive control panels, in which we started with CSF diluted at 1:2. The patient’s CSF ab titers were determined by fourfold serial dilutions; the ab reactivity remained positive at 1:2,048 under ×10 or ×20 objective lens (**Figure 2A**) but was negative at 1:8,192. Accordingly, the CSF ab titers were determined to be 1:2,048 as the greatest dilution at which a detectable positive result is still obtained with the fourfold serial dilution method.

We made a pair of positive control panels to score the intensity of ab reactivity (**Figures 2A, B** for CBA and IHC, respectively). A series of photographs was taken with the Olympus BX53 fluorescence microscope in several settings by adjusting the ISO/Exposure as follows: in panel A, the photographs were taken under the two settings of ISO/Exposure (1,600/500 ms and 800/500 ms) when viewing the slide under ×4, ×10, and ×20 objective lens. In panel B, the photographs were taken under the three settings of ISO/Exposure adjusted for each magnification of the objective lens as follows: 1,600/500 ms for ×4, 800/500 ms for ×10, and 400/500 ms for ×20, respectively. We used these several ISO/Exposure settings because it is difficult to score based on a single setting due to concomitant halation caused by the reactivity of antinuclear antibodies when present.

#### 2.3.2 Scoring of the intensity of antibody reactivity

We scored the intensity of ab reactivity of the individual patients’ CSF diluted at 1:2 on a scale from 0 to 6 (with ≥1 considered positive) visually based on the positive control panels. Through this scoring strategy, CSF ab titers can be estimated as follows: when the intensity of a patient’s CSF diluted at 1:2 is nearly identical to the score 6 (intensity of patient H’s CSF diluted at 1:2), the CSF ab titers can be estimated to be approximately 1:2,048 because patient H’s CSF ab titers are 1:2,048. In other words, “the intensity identical to the score 6” means that the patient’s CSF would be positive when the CSF is diluted five times in a fourfold serial dilution method (at 1: 2,048) but would be negative when being diluted six times (at 1:8,192). When the intensity of a patient’s CSF diluted at 1:2 is scored 3, it means that the patient’s CSF would still be positive when being diluted two times in a fourfold serial dilution method (at 1:32) but would be negative when being diluted three times (at 1:128), indicating that the estimated titers are 1:32. The intensity of ab reactivity that appeared higher than the score 6 was also scored 6 in this study. Accordingly, it can be considered as follows: the scores 6, 5, 4, 3, 2, and 1 correspond to estimated titers of 1:2,048 or more, 1:512, 1:128, 1:32, 1:8, and 1:2, respectively when CSF diluted at 1:2 is used. When no apparent reactivity was seen even when viewing under a ×20 objective lens, the score was considered to be 0.

HIS score was first determined by four authors (MI, NN, NK, and TI) independently and was finally determined by agreement of the authors on a scale from 0 to 6; however, when considered to be appropriate, a score of 0.5, 1.5, 2.5, 3.5, or 4.5 was given, but no sample scored 5.5 was seen in our cohort.

#### 2.3.3 Inter-assay reliability

We performed immunostaining twice in 21 patients’ CSF samples, which were selected from 79 GluN1-ab-positive patients in order to see an agreement of the score on each assay at different score levels (range, 0–6). The agreement between the scores of the first and second assay was evaluated using linear regression analysis.

#### 2.3.4 Evaluation of clinical features and HIS score of CSF obtained at diagnosis

In this study, we defined “pretreatment CSF” as CSF obtained before initiation of immunotherapy, while “posttreatment CSF” was defined as CSF after initiation of immunotherapy. We scored the intensity of CSF obtained from 79 patients (54 female, 68.3%), median age at onset 31 years (range, 12–74 years), whose CSF tested positive for GluN1-abs at Dalmau Lab. After reasonable exclusion of 11 patients (**Figure 1**), we assessed the score in the following three groups: group I was the primary group (*n* = 42), in which pretreatment CSF obtained within 4 weeks of encephalitis symptom (E-symptom) onset was available. The time from E-symptom onset to CSF collection was median 7 days (range, -1 to 28 days). We also assessed in the other two groups: group II (*n* = 50, with CSF obtained within 4 weeks of E-symptom onset regardless of pretreatment or posttreatment CSF) and group III (*n* = 51, with pretreatment CSF obtained within 3 months of E-symptom onset) (**Figure 1**). The subjects in group I were also included in groups II and III. The time from E-symptom onset to CSF collection in groups II and III was median 9 days (range, -1 to 28 days) and median 10 days (range, -1 to 90 days), respectively.

In general, it is ideal to evaluate pretreatment CSF to exclude a potential effect of immunotherapy on CSF ab titers at diagnosis. In a clinical setting, however, the pretreatment CSF is not always available. Furthermore, CSF ab titers may not immediately decline after initiation of immunotherapy due to sustained high disease activity. Therefore, we investigated the score in group II, including posttreatment CSF obtained during the acute stage. We also investigated the score in group III to include a small group of patients whose symptoms gradually developed beyond 4 weeks of E-symptom onset.

Immunotherapy included first-line therapy [intravenous high-dose methylprednisolone (IVMP), immunoglobulins, and plasma exchanges], second-line therapy (intravenous cyclophosphamide and rituximab), or other immunosuppressive drugs. E-symptoms were defined as those directly attributed to encephalitis, such as [1] abnormal (psychiatric) behavior or cognitive dysfunction (memory or psychobehavioral alterations), [2] speech dysfunction, [3] seizures, [4] movement disorder, dyskinesias, or rigidity/abnormal postures, [5] decreased level of consciousness, or [6] autonomic symptoms/central hypoventilation, which are listed as six core symptoms in the diagnostic criteria for anti-NMDAR encephalitis (6). Headache or fever that developed before E-symptom onset was not included in E-symptoms but was regarded as a preceding symptom. The clinical phenotype was evaluated by the sum of the number of the six core symptoms; a typical spectrum was defined as a manifestation with four or more core symptoms, while an incomplete phenotype was defined as a manifestation with three or fewer core symptoms, in which “isolated psychosis” is included.

We evaluated an association between the HIS score and a variety of clinical and paraclinical features, including sex, age at onset, the presence of tumor, preceding headache or fever, core symptoms, CSF total leukocyte count, CSF leukocyte count >5 cells/µL or >20 cells/µL, detection of oligoclonal bands (OCBs), elevated IgG index (≥0.74), brain MRI features suggestive of encephalitis, need for mechanical ventilation support, anti-NMDAR encephalitis one-year functional status (NEOS) score (21), worst functional status within 3 months of E-symptom onset, and 1-year functional status. The 1-year functional status was measured by modified Rankin Scale (mRS), in which good status was defined as mRS 0–2, while poor status was defined as mRS 3–6. Worst functional status was also measured by mRS. We defined brain MRI features suggestive of encephalitis as increased T2/FLAIR signal highly restricted to one or both medial temporal lobes or in multifocal areas involving gray matter, white matter, or both compatible with demyelination or inflammation, as defined in the possible AE diagnostic criteria (6).

### 2.4 Standard protocol approvals, registrations, and patient consents

The study was approved by the Institutional Review Board of Kitasato University (B20-280). Written informed consent was obtained from the patients or their proxies.

### 2.5 Statistical analysis

Statistical analyses were performed using JMP, version 17.0.0 (SAS Institute Inc.). Fisher exact test was performed for a comparison of categorical variables, and Wilcoxon test was used for continuous variables. Wilcoxon test with Bonferroni correction was used to compare worst mRS and NEOS score within each group. Spearman’s rank correlation was used to examine the relationship between continuous variables, but the inter-score agreement between the first and second assay was evaluated using linear regression analysis. Nominal logistic regression models were used to determine an association between HIS score at diagnosis and 1-year functional status or need for mechanical ventilation support. The statistical significance was set at *p* < 0.05. The sensitivity and the specificity of the commercial IHC were also determined with two-way contingency table analysis using JMP.

## 3 Results

### 3.1 Part I. Sensitivity and specificity of commercial fixed CBA

False-negative and false-positive results were seen in five of 79 ab-positive patients (6.3%) and four of 291 ab-negative patients (1.4%), respectively (**Table 1**). Although CSF ab titers were not determined at Dalmau’s Lab, according to the report, none of the five false-negative patients had high CSF ab titers: weakly positive in three (#1, 2, and 3), mildly positive in one (#4), and extremely very low in one (#5). These patients’ clinical phenotypes were also different from a typical spectrum of anti-NMDAR encephalitis: multifocal demyelinating syndrome (#1, later diagnosed with multiple sclerosis based on the subsequent course of the disease and the absence of GluN1-abs in the follow-up CSF), isolated seizures (#2), autoimmune post-herpes simplex encephalitis (#3), new-onset nonconvulsive status epilepticus (#4), and GlyR-ab-positive progressive encephalomyelitis with rigidity and myoclonus (#5).

**Table 1:** Results of GluN1 antibody testing.

	GluN1-antibody positive at Dalmau Lab (n = 79)	GluN1-antibody negative at Dalmau Lab (n = 291)
**GluN1-antibody positive** **on commercial CBA (n=78)**	True positive (n=74)	False positive (n=4)
**GluN1-antibody negative** **on commercial CBA (n=292)**	False negative (n=5)	True negative (n=287)

GluN1 antibodies were examined with commercial fixed CBA (EUROIMMUN AG, product No: FA 111m-3, Lübeck, Germany) (see Text) using CSF diluted at 1:2. The positivity or negativity of GluN1 antibodies was determined based on the results performed at the laboratory of Josep Dalmau (Barcelona).

In the four false-positive patients, HIS score was low (range, 1–1.5; estimated titers, 1:2–1:4), and their clinical features were inconsistent with anti-NMDAR encephalitis. Their final diagnosis included leptomeningeal metastasis of embryonal carcinoma (*n* = 1), overlapping NS-ab-negative encephalitis and MOG-ab-negative demyelinating syndrome (*n* = 1), GFAP-ab-positive encephalitis (*n* = 1), and probable neurodegenerative dementia with titin- and AchR-ab-positive myasthenia gravis (*n* = 1).

The sensitivity and the specificity of the commercial CBA were 93.7% (95% CI: 86.0–97.3) and 98.6% (95% CI: 96.5–99.5), respectively. The positive predictive value and negative predictive value were 94.9% (95% CI: 87.5–98.0) and 98.3% (95% CI: 96.1–99.3), respectively.

### 3.2 Part II. Clinical features, HIS score, inter-assay reliability, and clinical significance

#### 3.2.1 Clinical and paraclinical features in each group

The clinical and paraclinical features are summarized in **Table 2**. These features overlapped among the three groups because of the overlapping subjects. Female patients account for 73%–74% with a median age at onset of approximately 30 years. Preceding headache and fever were seen in 53%–56% and 47%–52% of patients, respectively. A typical spectrum was seen in 68%–69%. Among the six core symptoms, memory or psychobehavioral alterations were most frequently seen, followed by seizures, decreased level of consciousness, dyskinesias or associated movement disorders, speech dysfunction, and autonomic symptoms/central hypoventilation (**Table 2**). Mechanical ventilation support was needed in 51%–58%. Brain MRI features suggestive of encephalitis was seen in 39%–46%, CSF pleocytosis (>5 cells/µL) in 86%–94%, CSF pleocytosis (>20 cells/µL) in 61%–69%, OCBs in 62%–73%, and elevated IgG index in 30%–33%. Tumors were seen in 45%–50%; among those, ovarian teratoma (OT) was most frequent (49%–60% of female patients). High NEOS score (4 to 5) was seen in 20%–21%, while worst functional status (mRS 5 to 6) was observed in 80%–82%. Poor 1-year functional outcome was found in 21%–29%. First-line and second-line immunotherapy were used in 96%–98% and 47%–52%, respectively. Approximately 55% of patients did not show a clinical improvement within 4 weeks after initiation of immunotherapy or tumor removal. The HIS score in each group is also shown.

**Table 2:** Clinical and paraclinical features in each group.

	Group I (n=42)	Group II (n=50)	Group III (n=51)
**Female, n (%)**	31 (73.8)	37 (74.0)	37 (72.5)
**Age at onset (y), median (IQR, range)**	30.5 (21.8-38.3, 14-66)	29.5 (20.8-37, 14-66)	31.0 (24-39, 14-72)
**CSF obtained within 4 weeks of E-symptoms^1^ onset, n (%)**	42 (100)	50 (100)	42 (82.4)
**From E-symptom onset to CSF collection (days), median (IQR, range)**	7 (5-13, -1^2^-28)	9 (5-14, -1^2^-28)	10 (5-18, -1^2^-90)
**Pretreatment CSF, n (%)**	42 (100%)	42 (84%)	51 (100%)
**Headache that preceded E-symptom onset, n (%)**	22/40 (55.0)	27/48 (56.3)	26/49 (53.1)
**Fever that preceded E-symptom onset, n (%)**	22 (52.4)	25 (50.0)	24 (47.1)
**Typical spectrum^3^ (≥ 4 of 6 core symptoms), n (%)**	29 (69.0)	34 (68.0)	35 (68.6)
**Abnormal (psychiatric) behavior or cognitive dysfunction, n (%)**	41 (97.6)	48 (96.0)	50 (98.0)
**Speech dysfunction, n (%)**	27 (64.3)	34 (68.0)	30 (58.8)
**Seizures, n (%)**	34 (81.0)	41 (82.0)	41 (80.4)
**Movement disorder, dyskinesias, or rigidity/abnormal postures, n (%)**	27 (64.3)	32 (64.0)	30 (58.8)
**Decreased level of consciousness, n (%)**	32 (76.2)	37 (74.0)	38 (74.5)
**Autonomic symptoms/central hypoventilation, n (%)**	27 (64.3)	32 (64.0)	29 (56.9)
**Need for mechanical ventilation support, n (%)**	24 (57.1)	29 (58.0)	26 (51.0)
**Brain MRI features suggestive of encephalitis, n (%)**	19 (45.2)	23 (46.0)	20 (39.2)
**CSF leukocyte count (/µL), median (IQR, range)**	40 (14.8-122.3, 1-567)	43 (14.8-133, 1-567)	30 (12-105, 0-567)
**CSF leukocyte count > 5 cells/µL, n (%)**	39 (92.9)	47 (94.0)	44 (86.3)
**CSF leukocyte count > 20 cells/µL, n (%)**	29 (69.0)	34 (68.0)	31 (60.8)
**Detection of oligoclonal bands, n (%)**	27/37 (73.0)	32/44 (72.7)	28/45 (62.2)
**Elevated IgG index (≥0.74), n (%)**	13/39 (33.3)	15/46 (32.6)	14/47 (29.8)
**Presence of tumor including teratoma, n (%)**	19^4^ (45.2)	25^5^ (50.0)	24^6^ (47.1)
**Presence of ovarian teratoma in female patients, n (%)**	16/31 (51.6)	22/37 (59.5)	18/37 (48.6)
**High NEOS score (4-5), n (%)**	9 (21.4)	10 (20.0)	10 (19.6)
**Worst functional status (mRS 5-6)^7^, n (%)**	34 (81.0)	40 (80.0)	42 (82.4)^8^
**Poor one-year functional status, n (%)**	10/39 (25.6)	10/47 (21.3)	13/45 (28.9)
**First-line immunotherapy, n (%)**	41 (97.6)	49 (98.0)	49 (96.1)
**Second-line immunotherapy, n (%)**	22 (52.4)	26 (52.0)	24 (47.1)
**Lack of clinical improvement within 4 weeks after treatment, n (%)**	23/41 (56.1)	27/49 (55.1)	27/50 (54.0)
**HIS score at diagnosis, median (IQR, range)**	4 (3-6, 0-6)	4 (3-5.3, 0-6)	4 (3-5, 0-6)

Group I is a primary group, in which pretreatment CSF obtained within 4 weeks of E-symptom onset is available. Group II is a second group, in which CSF obtained within 4 weeks of E-symptom onset regardless of pretreatment or posttreatment CSF is available. Group III is a third group, in which pretreatment CSF obtained within 3 months of E-symptom onset is available. The subjects in Group I are all included in Groups II and III (see text).

^1^E-symptoms: encephalitis symptoms; ^2^ In one patient, CSF obtained one day before E-symptom onset was used; ^3^Typical spectrum is defined as a manifestation with four or more of the six core symptoms of anti-NMDAR encephalitis (see Text); ^4^Tumors included ovarian teratoma (n=16, 84.2%), suspected small cell lung cancer (SCLC) (n=1), esophageal carcinoma (n=1) and thyroid cancer (n=1). ^5^Tumors included ovarian teratoma (n=22, 88.0%), suspected SCLC (n=1), esophageal cancer (n=1), and thyroid cancer (n=1). ^6^Tumors included ovarian teratoma (n=18, 75.0%), suspected SCLC (n=1), SCLC (n=1), esophageal cancer (n=1), thyroid cancer (n=1), breast cancer (n=1) and large ovarian cyst (no teratoma was found pathologically) (n=1). ^7^Worst functional status within 3 months of E-symptom onset; ^8^One patient with SCLC who died within 3 months of E-symptom onset is included.

#### 3.2.2 HIS score at diagnosis in each group

**Figure 3:**
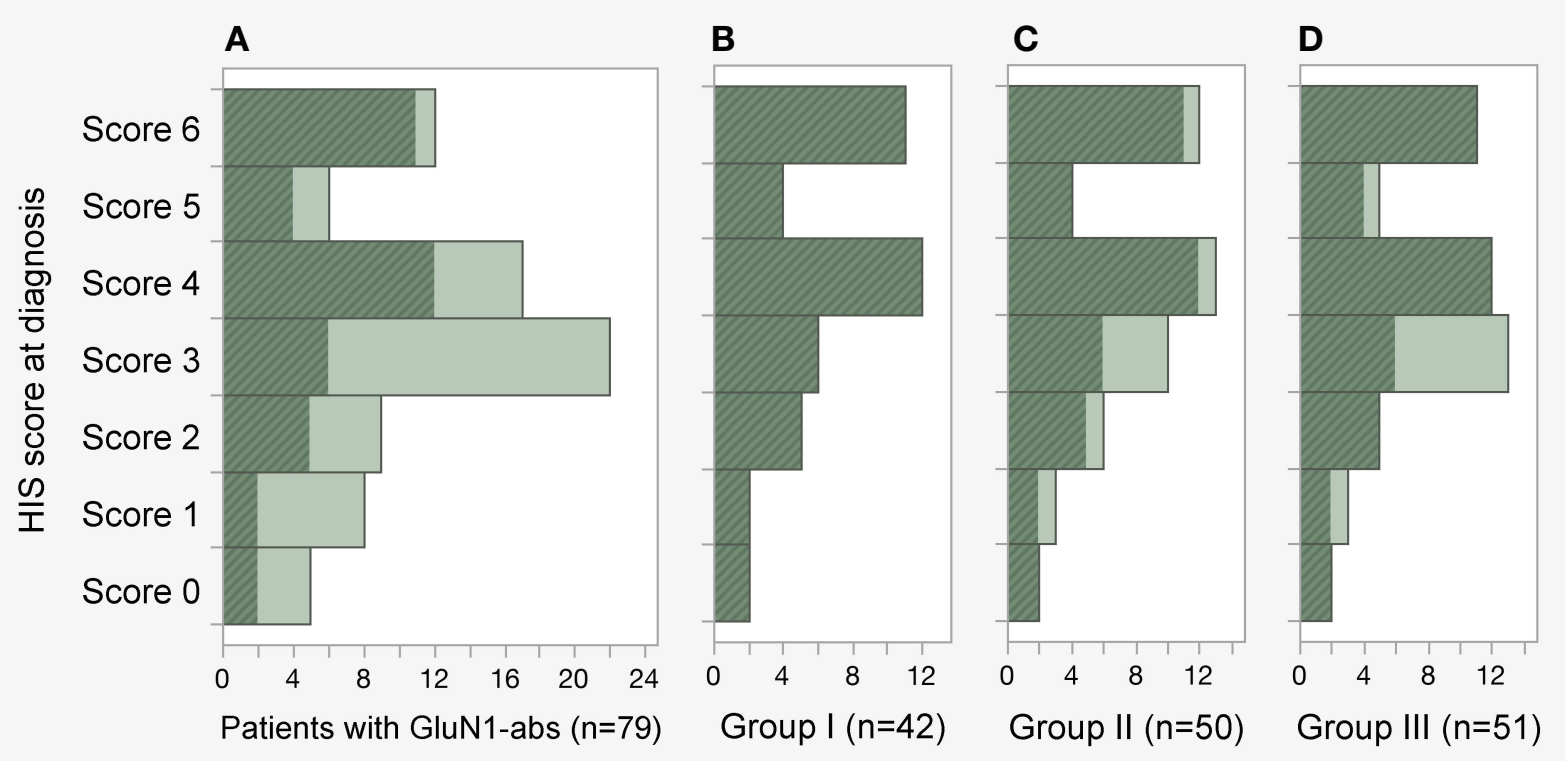
Distribution of H-intensity scale (HIS) score. **(A–D)** The HIS score in the 79-ab-positive patients, groups I, II and III was median 3.5 (IQR 2.5–4.5, range 0–6), 4 (IQR 3–6, range 0–6), 4 (IQR 3–5.3, range 0–6), and 4 (IQR 3–5, range 0–6), respectively. Note that the HIS score was determined using pretreatment cerebrospinal fluid (CSF) obtained within 4 weeks of E-symptom onset in group I **(B)**, CSF obtained within 4 weeks of E-symptom onset regardless of pretreatment or posttreatment CSF in group II **(C)**, and pretreatment CSF obtained within 3 months of E-symptom onset in group III **(D)**. Patients included in group I are shown with a diagonal stripe pattern in dark color. In the panels, patients who scored 4.5, 3.5, 2.5, and 1.5 were included in the histogram scored 4, 3, 2, and 1, respectively. abs, antibodies; E-symptom, encephalitis symptom; IQR, interquartile range.

HIS score at diagnosis is shown in **Figure 3**. In 79 ab-positive patients (**Figure 3A**), the HIS score was median 3.5 (interquartile range, IQR: 2.5–4.5; range, 0–6); five patients (6.3%) who scored 0 are false-negative (**Table 1**), but these samples are considered to have low or extremely low titers (see “Result”). Two of the five false-negative patients (#2 and 4) were also included in all three groups (**Figures 3B–D**). The median HIS score was 4 in each group, indicating that median CSF ab titers were estimated to be approximately 1:128.

#### 3.2.3 Inter-assay reliability of HIS score

There was a good agreement between the scores of the first and second assays (*β* = 0.9657, 95% CI = 0.9120–1.0195, *p* <.0001, *α* = 0.1044, 95% CI = -0.0876–0.2965, *p* = 0.2692, *R* ^2 =^ 0.9867; **Supplementary Figure S1**).

#### 3.2.4 Clinical significance of HIS score at diagnosis

**Figure 4:**
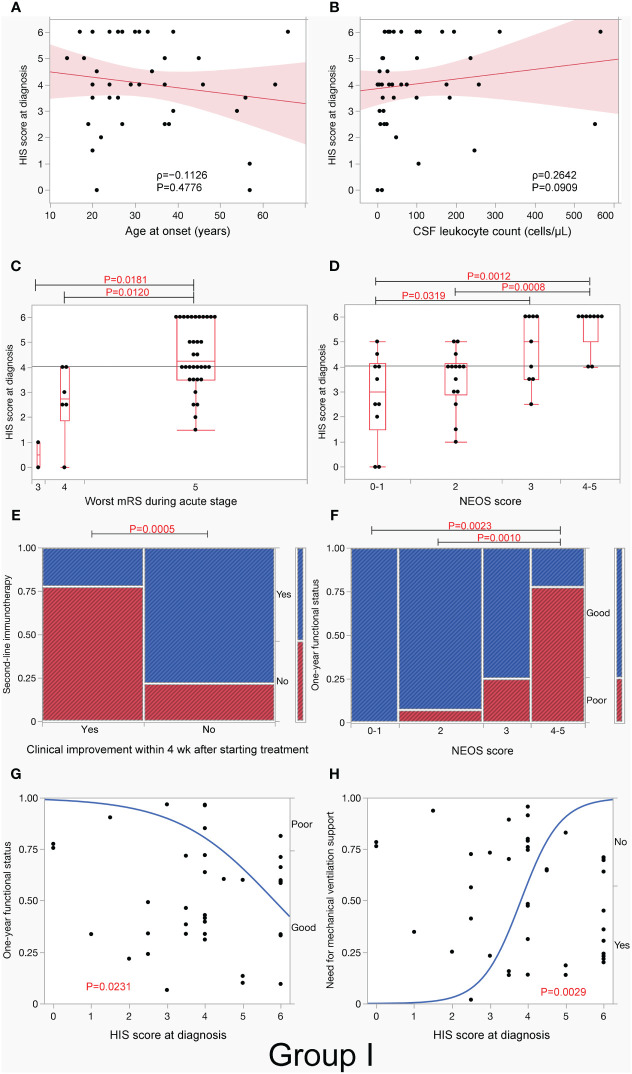
H-intensity scale (HIS) score and clinical/paraclinical features in group I. This figure shows an association between HIS score and clinical/paraclinical data, including age at onset **(A)**, CSF leukocyte count **(B)**, worst functional status within 3 months of E-symptom onset **(C)**, NEOS score **(D)**, use of second-line immunotherapy in patients with and without clinical improvement within 4 weeks after starting treatment **(E)**, 1-year functional status **(F, G)**, and need for mechanical ventilation support **(H)** in group I. Note that HIS score was higher in patients with worst functional status (mRS 5) **(C)** or high NEOS score (4 to 5) **(D)** than in those without, while HIS score had a significant effect on both 1-year functional status and need for mechanical ventilation support. In **(A, B)**, the red line represents the regression line, and the light red area represents the 95% CI of the line. In **(C, D)**, boxplots depict median and interquartile range with whiskers extending to minimum and maximum values. E-symptom, encephalitis symptom.

In group I, the median HIS score was not associated with sex, age at onset (**Figure 4A**), or CSF total leukocyte count (**Figure 4B**), but it was higher in patients with a typical spectrum than in those with an incomplete phenotype (*p* <.0001) (**Table 3**). The median HIS score was also higher in patients with a need for mechanical ventilation support (*p* <.0001), autonomic symptoms/central hypoventilation (*p* = 0.0002), dyskinesias (*p* = 0.0004), speech dysfunction (*p* = 0.0010), decreased level of consciousness (*p* = 0.0012), preceding headache (*p* = 0.0161), OT (*p* = 0.0211), and CSF leukocyte count > 20 cells/µL (*p* = 0.0278) than in those without, but there was no difference between patients with and without memory or psychobehavioral alterations, seizure, tumors, preceding fever, MRI features suggestive of encephalitis, CSF leukocyte count >5 cells/µL, OCB-detection, or elevated IgG index.

**Table 3:** Median HIS score (IQR, range) in each group.

	Group I	p value	Group II	p value	Group III	p value
**Sex** **Female** **Male**	4 (3.5-7, 0-6) 4 (2.5-4.5, 0-6)	0.1854	4 (3.3-6, 0-6) 3.5 (1.5-4.3, 0-6)	0.0746	4 (3.5-6, 0-6) 3.5 (2.9-4.1, 0-6)	0.1548
**Clinical phenotype^1^** **Typical spectrum (≥ 4 of 6 core symptoms)** **Incomplete phenotype (< 4 core symptoms)**	5 (4-6, 1.5-6) 2.5 (1.5-3.8, 0-4)	<.0001	4.5 (3.9-6, 1-6) 2.8 (2-3.4, 0-4)	<.0001	4.5 (3.5-6, 1.5-6) 2.8 (1.3-3.5, 0-4)	<.0001
**Abnormal (psychiatric) behavior or cognitive dysfunction** **Yes** **No**	4 (3.3-6, 0-6) 2.5 (2.5, 2.5)	0.2572	4 (3-5.8, 0-6) 2.3 (2-2.5, 2-2.5)	0.0833	4 (3.4-5, 0-6) 2.5 (2.5, 2.5)	0.2418
**Speech dysfunction** **Yes** **No**	4.5 (4-6, 2.5-6) 3 (1.5-4, 0-6)	0.0010	4 (3.5-6, 1-6) 3 (1.6-4, 0-6)	0.0019	4.5 (3.9-6, 2.5-6) 3.5 (1.8-3.8, 0-6)	0.0001
**Seizures** **Yes** **No**	4 (3.4-6, 0-6) 3.5 (2.6-4, 1-5)	0.0841	4 (3-6, 0-6) 3.5 (2.8-4, 1-5)	0.1153	4 (3.5-6, 0-6) 3.5 (2.1-4.3, 1-5)	0.1360
**Movement disorders^2^** **Yes** **No**	5 (4-6, 1.5-6) 3 (2-4, 0-5)	0.0004	4.5 (3.5-6, 1-6) 3 (2-4, 0-5)	0.0002	4.8 (3.5-6, 1.5-6) 3.5 (2.3-4, 0-5)	<.0001
**Decreased level of consciousness** **Yes** **No**	4.5 (3.6-6, 1.5-6) 2.8 (0.8-4, 0-4)	0.0012	4 (3.5-6, 1-6) 3 (1.5-3.8, 0-4)	0.0004	4 (3.5-6, 1.5-6) 3 (1-3.8, 0-4)	0.0003
**Autonomic symptoms/central hypoventilation** **Yes** **No**	5 (4-6, 1.5-6) 3 (2-4, 0-4)	0.0002	4.8 (3.5-6, 1-6) 3 (2-4, 0-4)	0.0001	5 (3.5-6, 1.5-6) 3.5 (2.4-4, 0-4)	0.0001
**Need for mechanical ventilation support** **Yes** **No**	5 (4-6, 2.5-6) 3 (1.9-4, 0-4)	<.0001	5 (4-6, 1-6) 3 (2-4, 0-4)	<.0001	5 (4-6, 2.5-6) 3.5 (2.3-4, 0-5)	<.0001
**Headache that preceded E-symptom onset** **Yes** **No**	4.8 (4-6, 1.5-6) 3.5 (2.4-4.3, 0-6)	0.0161	4.5 (3.5-6, 1-6) 3.5 (2.3-4, 0-6)	0.0137	4.5 (3.5-6, 1.5-6) 3.5 (2.5-4, 0-6)	0.0067
**Fever that preceded E-symptom onset** **Yes** **No**	4.3 (2.5-6, 0-6) 4 (3.1-5, 0-6)	0.7103	4 (2.5-6, 0-6) 4 (3-5, 0-6)	0.7529	4.3 (2.8-6, 0-6) 3.5 (3-4, 0-6)	0.2972
**Brain MRI features suggestive of encephalitis** **Yes** **No**	4 (3.1-6, 0-6) 4.3 (2.5-5.3, 0-6)	0.7781	4 (3-6, 0-6) 4 (2.5-5, 0-6)	0.9843	4 (3.3-6, 0-6) 3.8 (2.9-5, 0-6)	0.5412
**CSF leukocyte count > 5 cells/µL** **Yes** **No**	4 (3-6, 0-6) 4 (0-4, 0-4)	0.2854	4 (3-6, 0-6) 4 (0-4, 0-4)	0.3957	4 (3.1-5.8, 0-6) 3.5 (1-4, 0-4)	0.0758
**CSF leukocyte count > 20 cells/µL** **Yes** **No**	4 (3.5-6, 1-6) 3.5 (2.5-4.3, 0-5)	0.0278	4 (3.5-6, 1-6) 3.3 (2.1-4, 0-5)	0.0089	4 (3.5-6, 1-6) 3.5 (2.6-4, 0-5)	0.0028
**Detection of oligoclonal bands** **Yes** **No**	4 (3.5-6, 1-6) 3.3 (1.5-4.6, 0-6)	0.0847	4 (3.5-6, 1-6) 3 (2.1-4.4, 0-6)	0.0477	4 (3.5-6, 1-6) 3.5 (2.3-4, 0-6)	0.0069
**Elevated IgG index** **Yes** **No**	5 (3.8-6, 1-6) 4 (2.9-5, 0-6)	0.1537	5 (3.5-6, 1-6) 4 (3-4.5, 0-6)	0.0616	5 (1.3-6, 1-6) 3.5 (3-4.5, 0-6)	0.0432
**Systemic tumors including ovarian teratoma** **Yes** **No**	5 (3.5-6, 0-6) 4 (2.5-6, 0-6)	0.0538	4 (3.5-6, 0-6) 4 (2.5-4.3, 0-6)	0.0602	4.3 (3.5-6, 0-6) 3.5 (3-4, 0-6)	0.0869
**Ovarian teratoma** ** Yes** **No**	5 (4-6, 2-6) 4 (2.5-4.7, 0-6)	0.0211*	4.3 (3.5-6, 2-6) 3.8 (2.5-4.4, 0-6)	0.0331*	4.8 (3.5-6, 2-6) 3.5 (2.8-4.3, 0-6)	0.0170*
**Clinical improvement ≤ 4 weeks of treatment initiation** ** No** **Yes**	5 (4-6, 1.5-6) 3.8 (2.4-4.1, 0-5)	0.0016	5 (3.5-6, 1-6) 3.3 (2.4-4, 0-5)	0.0008	5 (3.5-6, 1-6) 3.5 (2.5-4, 0-5)	0.0014
**Need for ICU admission** **Yes** **No**	5 (4-6, 2.5-6) 3 (1.8-4, 0-4)	<.0001	5 (4-6, 1-6) 3 (2-4, 0-4)	<.0001	5 (3.8-6, 2.5-6) 3.3 (1.9-4, 0-4)	<.0001
**Second-line immunotherapy** **Yes** **No**	4 (4-6, 1.5-6) 3.5 (2.5-5, 0-6)	0.0500	4 (3.9-6, 1.5-6) 3.3 (2.1-4.9, 0-6)	0.0108	4 (3.6-6, 1.5-6) 3.5 (2.5-5, 0-6)	0.0212

Group I is a primary group, in which pretreatment CSF obtained within 4 weeks of E-symptom onset is available. Group II is a second group, in which CSF obtained within 4 weeks of E-symptom onset regardless of pretreatment or posttreatment CSF is available. Group III is a third group, in which pretreatment CSF obtained within 3 months of E-symptom onset is available.

^1^Typical spectrum is defined as a manifestation with four or more of the six core symptoms, while forme fruste is defined as an incomplete manifestation with three or fewer core symptoms.

^2^Movements disorders including dyskinesias or rigidity/abnormal postures. *P value was assessed in all patients including male patients.

CSF, cerebrospinal fluid; E-symptom, encephalitis symptoms.

The median HIS score was higher in patients with mRS of 5 than in those with mRS of 4 (*p* = 0.0120) or mRS of 3 (*p* = 0.0181) (**Figure 4C**). The median HIS score was also higher in patients with a high NEOS score (4 to 5) than in those with a low NEOS score (0 to 1) (*p* = 0.0012) or NEOS score 2 (*p* = 0.0008) (**Figure 4D**) and was higher in patients with need for ICU admission than in those without (*p* <.0001) and in patients who received second-line immunotherapy than in those who did not (*p* = 0.0500). The median HIS score was also higher in patients without clinical improvement within 4 weeks after initiation of treatment than in those with clinical improvement (*p* = 0.0016, **Table 3**). Such patients, who did not show clinical improvement within 4 weeks after initiation of treatment, more frequently received second-line immunotherapy than those who did (*p* = 0.0005, **Figure 4E**). Patients with a high NEOS score (4 to 5) had more frequently poor 1-year functional status than those with low NEOS score 2 (*p* = 0.0010) or score of 0 to 1 (*p* = 0.0023) (**Figure 4F**).

A logistic regression analysis revealed that HIS score at diagnosis had a mild but significant effect on 1-year functional status (*p* = 0.0231) (**Figure 4G**). However, there was no difference in 1-year functional status between patients with second-line immunotherapy and those without [13/21 (61.9%) vs. 16/18 (88.9%), *p* = 0.0740, not shown]. It was also shown that HIS score at diagnosis had also a significant effect on need for mechanical ventilation support (*p* = 0.0029), with a probability of 17% of mechanical ventilation support on score 3, 59% on score 4, and 91% on score 5, respectively (**Figure 4H**); a similar effect was seen in groups II and III (**Supplementary Figures S2F**, **S3F**).

Among the three groups, most of the results were similar, but there were a few differences (**Table 3**). In group II, the median HIS score was higher in patients with OCB-detection than in those without (*p* = 0.0477), while HIS score had a significant effect on 1-year functional outcome (*p* = 0.0126, **Supplementary Figure S2E**), as did in group I. In group III, the median HIS score was higher not only in patients with OCB-detection (*p* = 0.0069) but also in patients with elevated IgG index (*p* = 0.0432) than in those without (**Table 3**). In group III, HIS score did not have a significant effect on 1-year functional outcome (*p* = 0.0582, **Supplementary Figure S3E**). There was no association between the use of second-line immunotherapy and 1-year functional status in either group II (*p* = 0.0786) or III (*p* = 0.5136) (data not shown).

## 4 Discussion

This study demonstrates the following findings: (1) the commercial fixed CBA is a reliable assay with high sensitivity and high specificity, even when CSF diluted at 1:2 is used; (2) a good inter-assay agreement was seen; (3) HIS score at diagnosis was higher in patients with a typical spectrum of anti-NMDAR encephalitis, dyskinesias or associated movement disorders, decreased level of consciousness, autonomic symptoms/central hypoventilation, speech dysfunction, preceding headache, need for mechanical ventilation support, worst functional status (mRS 5 to 6), high NEOS score (4 to 5), OT, or CSF leukocyte count >20 cells/µL than those without, (4) the HIS score at diagnosis may be used to predict a probability of need for mechanical ventilation support, (5) higher GluN1-ab titers in CSF obtained at diagnosis may play a role in poor 1-year functional status, and (6) an incomplete phenotype can be attributed to low CSF ab titers.

We have developed this score to estimate CSF ab titers with one-time immunostaining and conducted this study to clarify whether (1) the score of the CSF obtained at diagnosis is associated with clinical/paraclinical features and (2) the HIS score can be used as a marker to predict the subsequent course of disease. The HIS score does not provide CSF ab titers directly measured by ELISA or using serial dilutions, but this strategy provides estimated CSF ab titers as an HIS score, ranging from 1:2 (score 1), 1:8 (score 2), 1:32 (score 3), 1:128 (score 4), and 1:512 (score 5) to 1:2,048 or more (score 6). The estimated CSF ab titers are close to those measured in a fourfold serial dilution method. The HIS score can be used as a marker not only of a disease activity but also of 1-year functional status, but individual laboratories have to make their own positive control panels. The ideal positive control patient’s CSF ab titers are unclear, but we used patient H’s CSF having ab titers of 1:2,048 and developed the visually distinguishable six-grade positive control panels.

After starting this study, however, we received another patient’s CSF, which revealed intense reactivity far beyond that of patient H’s CSF. The CSF ab titers were determined to be 1:32,768 in a fourfold serial dilution method. If we revised the positive control panels using this exceptionally high ab titer CSF, we could have developed a pair of eight-grade positive control panels (estimated CSF ab titers, 1:2–1:32,768). However, we did not revise the panels because it is likely to be difficult to recognize visually subtle difference in intensity between the scores 6 and 7 as well as 7 and 8, and we did not find out a clinical value that it is better to distinguish patients with extremely high CSF ab titers (>1:2,048) from those with CSF ab titers of 1:2,048. On the other hand, it is still possible to estimate CSF ab titers using these positive control panels even when a patient’s CSF ab titers are >1:2,048 through additional one-time immunostaining using the patient’s CSF diluted at 1:8,192. When the patient’s CSF diluted at 1:8,192 is scored 2, the CSF ab titers can be estimated to be approximately 1:32,768 because “the score 2” means that the patient’s diluted CSF would still be positive when being diluted one time in a fourfold serial dilution (diluted at 1:32,768) but would be negative when being diluted twice (diluted at 1:131,072), indicating that the CSF ab titers are estimated to be approximately 1:32,768.

Among the six core symptoms, HIS score was not associated with memory or psychobehavioral alterations or seizures. In our cohort, 96%–98% of patients presented with memory or psychobehavioral alterations. Accordingly, it was statistically difficult to compare the HIS score between those with and without memory or psychobehavioral alterations because of the too small sample size in one arm, whereas seizures were seen in 80%–82% of patients but less frequently than memory or psychobehavioral alterations. We did not find a significant difference in HIS score between patients with and without seizures. On bedside examination, it is often difficult to distinguish epileptic seizure from non-epileptic seizure or seizure from paroxysmal movement disorders, particularly in unresponsive patients with anti-NMDAR encephalitis. In most of them, these symptoms occur concurrently or one after another and are often indiscernible (3). Seizure was more frequently reported by referring physicians than dyskinesias or associated movement disorders (80%–82% vs. 59%–64%). Given the presence of a significant association between movement disorders and HIS score in all groups, high CSF ab titers play an important role in “dyskinesias or associated movement disorders” than in “seizure”. The ambiguity of the term “seizures” and its indiscernibility from paroxysmal movement disorders may lead to a lack of significant relationship between HIS score and seizures.

Memory or psychobehavioral alterations were not associated with HIS score; however, it is important to note that prominent psychobehavioral alterations are almost always seen in the early stage, and HIS score was significantly lower in patients with an incomplete phenotype than in those with a typical spectrum, indicating that “isolated psychosis” can be attributed to low CSF ab titers. It is also extremely important in such low-ab-positive patients with an incomplete phenotype to perform IHC adapted to NS antigens or live neurons to exclude false positive results and prevent unnecessary repeated immunotherapies in patients with a primary psychiatric disorder or non-immune-mediated epilepsy (22).

Regarding an association with a tumor, approximately half of the patients were found to have tumors (mostly OT); OT was found in approximately 50%–60% of female patients (**Table 2**). We found that the median HIS score was higher in patients with OT than in those without in all three groups, but it was not significantly higher in patients with tumors (when cancers were included; **Table 2**) than in those without in any group. The results of this study support the previous observation (8) that CSF ab titers were higher in patients with teratoma than in those without teratoma. It is suggested that teratoma plays an important role in ab production though NMDAR expressed in the nervous tissue contained in the teratoma, which are taken up by antigen-presenting cells and are presented to the immune system (23) or through ectopic germinal center formation in the teratoma (24).

We also investigated a possible association between HIS score and paraclinical findings. The HIS score was not associated with either OCB-detection or elevated IgG index in group I, but OCB-detection in groups II and III, and elevated IgG index in group III. In our cohort, OCBs were detected in two-thirds of the patients and elevated IgG index in one-third (**Table 2**). In group II, posttreatment CSF obtained after initiation of IVMP are included in 16% of patients; despite this, the HIS score was higher in patients with OCB-detection than those without. As intrathecal ab synthesis has been demonstrated in anti-NMDAR encephalitis (25), it is reasonable to think that high CSF ab titers are, in part, attributed to intrathecal ab synthesis, but elevated IgG index might be less sensitive than OCB-detection in terms of a biomarker of intrathecal ab synthesis. We also investigated an association between the HIS score and CSF leukocyte count in three variables: CSF total leukocyte count and CSF leukocyte count >5 cells/µL or >20 cells/µL. Among those, only CSF leukocyte count >20 cells/µL, which is an independent predictor for outcome in NEOS score (21), showed a significant association in all three groups. Brain MRI features suggestive of encephalitis was seen in 39%–46% of the patients, but the HIS score was not associated with MRI abnormalities, suggesting that factors other than high CSF ab titers, such as concurrent abs against MOG, AQP4, GFAP (19, 26), or other surface antigens not identified, or T-cell-mediated injury may contribute to MRI abnormalities. These glial surface antibodies were concurrently detected in some of the patients, including those with abnormal MRI findings but were not examined in all patients in our cohort. Extreme delta blush pattern was seen in some of the patients, but we did not investigate an association between the HIS score and EEG abnormalities because the EEG recorded during the course of the disease is not fully available for review.

The HIS score at diagnosis was higher in patients with high NEOS score (4 to 5), worst functional status (mRS 5 to 6), and need for mechanical ventilation support than those without in all three groups, and it had a significant effect on 1-year functional status in groups I and II, but not in group III. These data suggest that high CSF ab titers, particularly in the CSF obtained within 4 weeks of E-symptom onset, determine a disease severity and play an important role in the subsequent course of disease. These results are not inconsistent with the previous observations that high NEOS score (4 to 5) is associated with poor 1-year functional status (21), and high CSF ab titers are associated with poor functional outcome (7, 8). Accordingly, higher CSF ab titers at diagnosis are considered to play an important role in poor 1-year functional status but may not be a sole factor contributing to poor outcome. In most of the cases, CSF ab titers usually decline with time after initiation of immunotherapy, and functional recovery is associated with a reduction in CSF ab titers (7, 8); however, approximately 20% of cases are refractory to immunotherapy. In such refractory cases, sustained high CSF ab titers have been reported (8). Therefore, sustained high ab titers are likely to be a more important factor contributing to poor long-term functional status than the initial high ab titers.

In our cohort, we did not find a positive effect of the use of second-line immunotherapy on 1-year functional status in any group. However, patients who were treated with second-line immunotherapy exhibited a more frequently poor response to initial treatment than those who were not treated. Furthermore, the median CSF ab titers at diagnosis were higher in patients who were treated with second-line immunotherapy than in those who were not treated. Accordingly, the lack of positive effect on 1-year functional status does not exclude the efficacy of second-line immunotherapy.

This study has limitations because of it being a retrospective study, having a small sample size, and having analysis based on estimated CSF ab titers without serial dilutions. EEG findings are not included in this study. GFAP, MOG, and AQP4 abs were examined in some but not in all patients. Chronological changes in the HIS score are not included in the analysis. Despite these limitations, we showed an association between estimated CSF ab titers at diagnosis through the HIS score and some of the clinical/paraclinical features, disease severity, and 1-year functional status. A nominal logistic model may also help to predict a probability of need for mechanical ventilation support (**Figure 4H**, **Supplementary Figures S2F**, **S3F**). This strategy is relatively easy to determine CSF ab titers with only one-time immunostaining without serial dilutions at a low cost, can be used in clinical practice at any laboratory when minimum equipment for IIFA is available, and provides clinicians with important clues regarding this devastating but potentially reversible AE.

## Data availability statement

The original contributions presented in the study are included in the article/**Supplementary Material**. Further inquiries can be directed to the corresponding author.

## Ethics statement

The studies involving humans were approved by Institutional Review Boards of Kitasato University (B20-280). The studies were conducted in accordance with the local legislation and institutional requirements. Written informed consent for participation in this study was provided by the participants’ legal guardians/next of kin. The animal study was approved by Animal Experimentation and Ethics Committee of the Kitasato University School of Medicine (2023–069). The study was conducted in accordance with the local legislation and institutional requirements.

## Author contributions

MI: Conceptualization, Data curation, Investigation, Methodology, Writing – original draft, Writing – review & editing. NN: Conceptualization, Data curation, Investigation, Methodology, Writing – review & editing. NK: Conceptualization, Data curation, Investigation, Methodology, Writing – review & editing. TIw: Data curation, Writing – review & editing. MNag: Data curation, Writing – review & editing. MNak: Data curation, Writing – review & editing. JK: Data curation, Writing – review & editing. EK: Data curation, Writing – review & editing. KN: Data curation, Writing – review & editing. NM: Data curation, Formal analysis, Methodology, Writing – review & editing. TIi: Conceptualization, Data curation, Formal analysis, Funding acquisition, Investigation, Methodology, Project administration, Resources, Supervision, Visualization, Writing – original draft, Writing – review & editing.

## Supplementary material

Supplementary Figure 1 Inter-assay reliability. H-intensity scale score was determined twice in 21 patients’ cerebrospinal fluid samples with different GluN1 antibody titers that scored from 0 to 6. This figure reveals a good agreement between the scores of the first and second assays.

Supplementary Figure 2 H-intensity scale (HIS) score and clinical/paraclinical features in group II. HIS score was higher in patients with worst functional status within 3 months of E-symptom onset **(A)** and a high NEOS score (4 to 5) **(B)** than in those without. Second-line immunotherapy was more frequently used in patients who did not show clinical improvement within 4 weeks after starting treatment than in those who did **(C)**. Patients with a high NEOS score (4 to 5) more frequently had a poor 1-year functional status **(D)**. HIS score at diagnosis **(E)** had a significant effect on both 1-year functional status and need for mechanical ventilation support **(F)** (see **Table 2**). In **(A, B)**, boxplots depict median and interquartile range with whiskers extending to minimum and maximum values. E-symptom, encephalitis symptom.

Supplementary Figure 3 H-intensity scale (HIS) score and clinical/paraclinical features in group III. HIS score was higher in patients with worst functional status within 3 months of E-symptom onset **(A)** and a high NEOS score (4 to 5) **(B)** than in those without. Second-line immunotherapy was more frequently used in patients who did not show clinical improvement within 4 weeks after starting treatment than in those who did **(C)**. Patients with a high NEOS score (4 to 5) more frequently had a poor 1-year functional status compared with those without **(D)**. HIS score at diagnosis **(E)** did not have a significant effect on 1-year functional status in group III, but it had on need for mechanical ventilation support **(F)** (see **Table 2**). In **(A, B)**, boxplots depict median and interquartile range with whiskers extending to minimum and maximum values. E-symptom, encephalitis symptom.
