# Supplementary material for: H-intensity scale score to estimate CSF GluN1 antibody titers with one-time immunostaining using a commercial assay
Source: Front Immunol. 2024 Apr 30;15:1350837. doi: 10.3389/fimmu.2024.1350837 (PMC11091310; doi:10.3389/fimmu.2024.1350837)
Supplement: Supplementary Figure 1 — Inter-assay reliability. H-intensity scale score was determined twice in 21 patients’ cerebrospinal fluid samples with different GluN1 antibody titers that scored from 0 to 6. This figure reveals a good agreement between the scores of the first and second assays. [file Image_1.pdf]

Supplementary Material

Supplementary Figure 1: Inter-assay reliability

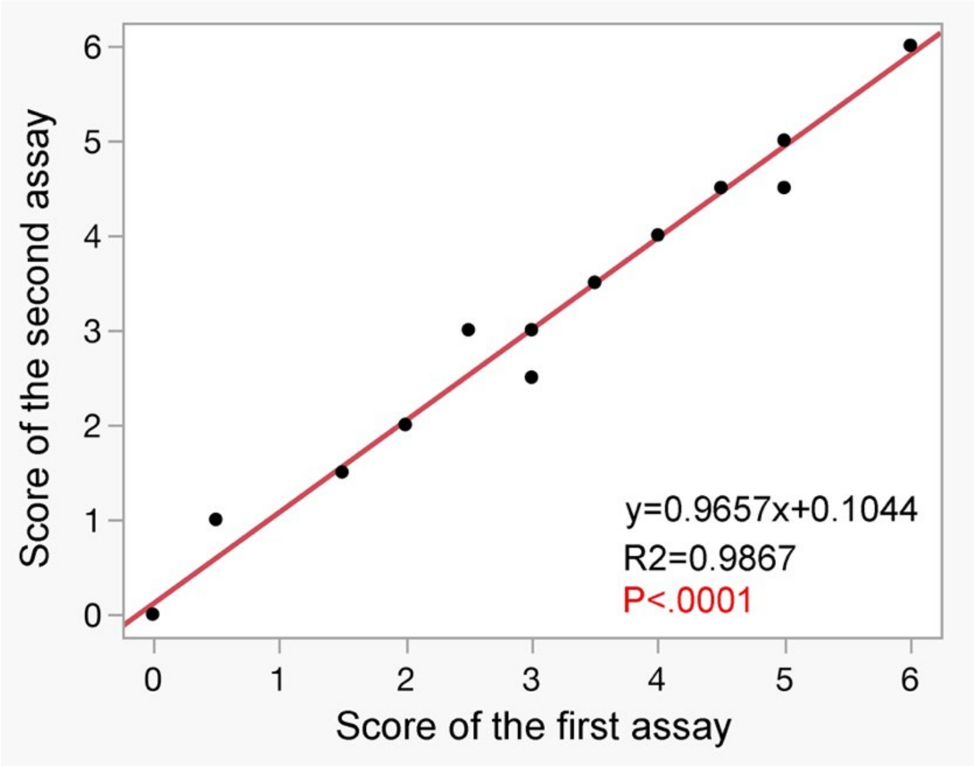

H-intensity scale (HIS) score was determined twice in 21 patients' CSF samples with different GluN1 antibody titers that scored from 0 to 6. This figure reveals a good agreement between the score of the first and second assay.
