# Supplementary material for: H-intensity scale score to estimate CSF GluN1 antibody titers with one-time immunostaining using a commercial assay
Source: Front Immunol. 2024 Apr 30;15:1350837. doi: 10.3389/fimmu.2024.1350837 (PMC11091310; doi:10.3389/fimmu.2024.1350837)
Supplement: Supplementary Figure 3 — H-intensity scale (HIS) score and clinical/paraclinical features in group III. HIS score was higher in patients with worst functional status within 3 months of E-symptom onset (A) and a high NEOS score (4 to 5) (B) than in those without. Second-line immunotherapy was more frequently used in patients who did not show clinical improvement within 4 weeks after starting treatment than in those who did (C). Patients with a high NEOS score (4 to 5) more frequently had a poor 1-year functional status compared with those without (D). HIS score at diagnosis (E) did not have a significant effect on 1-year functional status in group III, but it had on need for mechanical ventilation support (F) (see Table 2 ). In (A, B), boxplots depict median and interquartile range with whiskers extending to minimum and maximum values. E-symptom, encephalitis symptom. [file Image_3.pdf]

Supplementary Material

Supplementary Figure 3: HIS score and clinical/paraclinical features in Group III

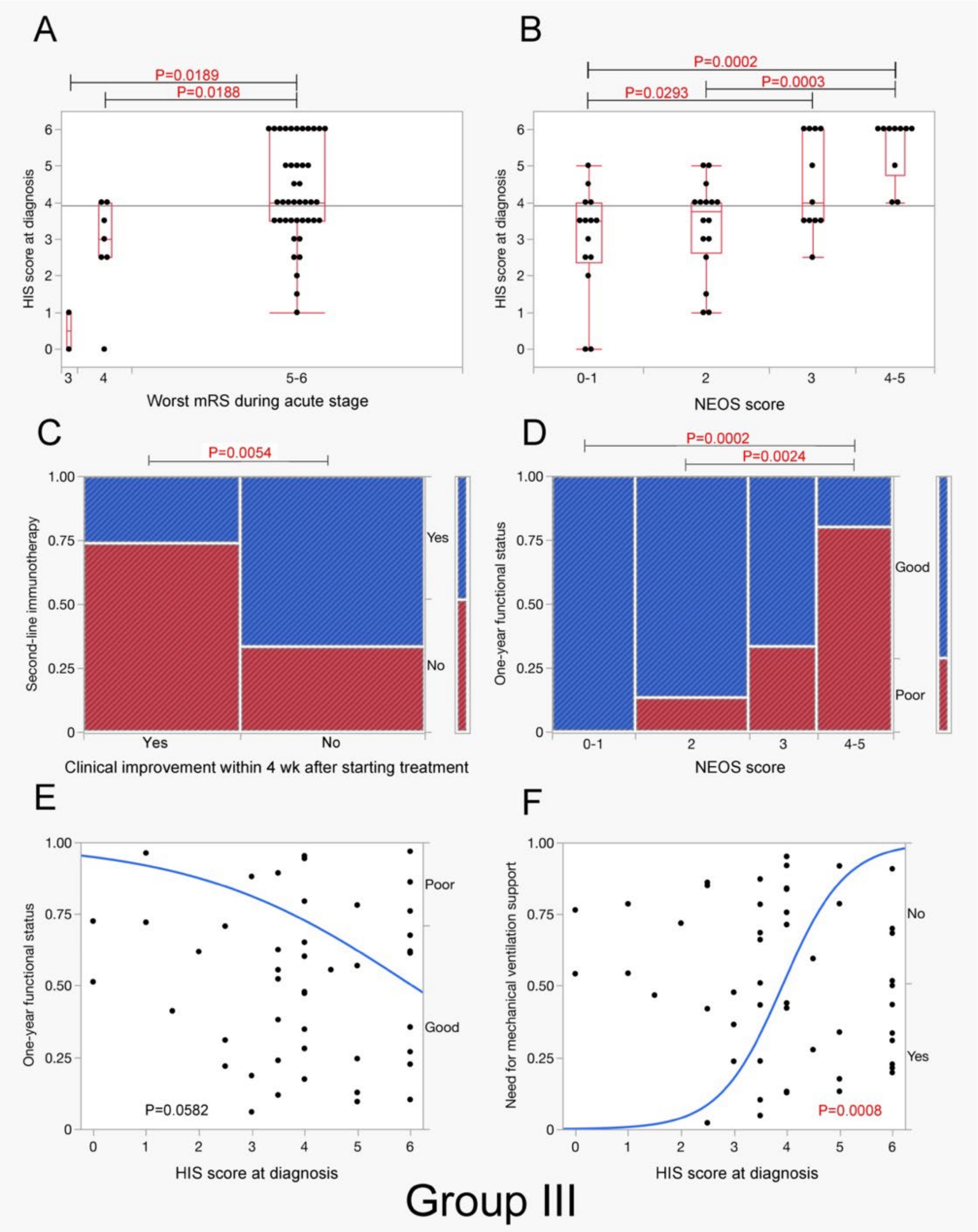

H-intensity scale (HIS) score was higher in patients with worst functional status within 3 months of E-symptom onset (A) and high NEOS score (4 to 5) (B) than in those without. Second-line immunotherapy was more frequently used in patients who did not show clinical improvement within 4 weeks after starting treatment than in those who did (C). Patients with a high NEOS score (4 to 5) more frequently had a poor 1-year functional status compared with those without (D). HIS score at diagnosis (E) did not have a significant effect on 1-year functional status in Group III, but it had on need for mechanical ventilation support (F). See Table 2. In (A) and (B), boxplots depict median and interquartile range with whiskers extending to minimum and maximum values. E-symptom: encephalitis symptom
